# β-Glucans, Triterpenes and Nucleoside Analogs of Edible and Medicinal Mushrooms as Complementary Treatment in Diabetes and Cancer—A Review of Evidence from Clinical Trials

**DOI:** 10.3390/cancers18142294

**Published:** 2026-07-16

**Authors:** Mariann Paulinné Bukovics, Laura Simon-Szabó, István Takács, Zsuzsanna Németh

**Affiliations:** 1 Independent Researcher, 1084 Budapest, Hungary; bukovics.mariann0710@gmail.com; 2 Department of Molecular Biology, Semmelweis University, Tuzoltó u. 37–47, 1094 Budapest, Hungary; szabo.laura@semmelweis.hu; 3 Department of Internal Medicine and Oncology, Semmelweis University, Koranyi S. u 2/a, 1083 Budapest, Hungary; takacs.istvan@semmelweis.hu

**Keywords:** edible mushrooms, medicinal mushrooms, diabetes, cancer, complementary therapy

## Abstract

There is a growing interest in preventive and complementary therapies to support health, increase the effectiveness of conventional medical treatments, and improve quality of life (QoL). This review summarizes the effects of β-glucans, triterpenes and nucleoside analogs of edible and/or medicinal mushrooms with results of clinical trials that investigated these effects in diabetes, breast-, prostate-, lung- and colorectal cancer patients. The results support that these components effectively alleviate symptoms and improve quality of life of these patients. Higher than 3 g of mushrooms, or equivalent extracts, per day are able to diminish metabolic parameters associated with diabetes, i.e., hyperglycemia, hyperlipidemia, and inflammation. The found survival benefit of mushrooms in breast cancer patients was subgroup- or marker-specific. Additionally, their immunomodulatory effects were more complex—different immunological parameters could be either activated or suppressed by different amounts. Data are limited for prostate cancer patients to date, but lentinan intake may increase the survival time of these patients. The survival of lung cancer patients is significantly improved with the use of beta-glucan as complementary therapy. However, its immunomodulatory role was not confirmed. The application of these mushroom components in patients with colorectal cancer may improve QoL. However, survival benefits are inconsistent across different clinical trials of these patients. In summary, although further randomized clinical trials are still required to evaluate optimal dosage and long-term mechanisms of actions in humans, edible and medicinal mushrooms may serve as valuable tools in preventive or in complementary therapies beside the conventional medical treatments of diabetes and cancer.

## Simple Summary

The effects of β-glucans, triterpenes and nucleoside analogs of edible and/or medicinal mushrooms with results from clinical trials investigating these effects in diabetes, breast-, prostate-, lung- and colorectal cancer patients have been summarized in this review. The findings confirm benefits in taking higher than 3 g mushroom powder daily (or equivalent extracts) to improve metabolic parameters in diabetic patients. Quality of life benefits were supported in all four cancer types across the clinical trials. Additionally, significantly improved survival times were found in marker- or subgroup-specific breast cancer patients, and also in patients diagnosed with lung and prostate cancers. Although further randomized clinical trials are still required to evaluate optimal dosage and long-term mechanisms of actions in humans, the results of this review support that edible and medicinal mushrooms can serve as valuable tools in preventive and/or in complementary therapies beside the conventional medical treatments of diabetes and cancer.

## 1. Introduction

Fungi constitute an independent kingdom in taxonomy, distinct from both plants and animals. Fungi have been utilized for thousands of years not only as food source but also as therapeutic agents [1,2]. An example of this is *Monascus purpureus*, commonly known as fermented red yeast rice; it has been an integral part of traditional Chinese culinary practices for over a thousand years. It has primarily been used to flavor, color, and preserve foods such as rice, tofu, and meats. At the same time, it has also been used in Traditional Chinese Medicine (TCM) to support digestion, improve blood circulation and resolve blood “stasis” [3].

Traditionally, edible mushrooms are primarily appreciated for their rich nutritional profile, especially because of their protein, dietary fiber and vitamin content. Although edible mushrooms, traditionally, are not counted as medicinal mushrooms, they also contain important bioactive compounds, including polysaccharides (i.e., β-glucans), phenolic compounds, sterols, and antioxidants [4]. Bioactive compounds are defined as either essential or non-essential molecules (e.g., vitamins or polyphenols) that occur in nature as part of the food chain and exert measurable effects of human health [5].

The bioactive compounds of those mushrooms which are consumed generally as food are present at lower concentrations than in the so-called “medicinal mushrooms”. It means medicinal mushroom species, whether collected in the wild or cultivated (selected and standardized) to be primarily used as food supplement, that contain higher concentrations and/or a broader spectrum of bioactive compounds [6,7,8]. For instance, *Pleurotus ostreatus* (Oyster mushroom), whether cultivated or foraged, contains bioactive compounds such as β-glucans and ergothioneine; however, their levels are lower compared to those medicinal species like *Ganoderma lucidum* and *Lentinula edodes* either cultivated or found in nature [9,10]. Nonetheless, edible mushrooms consumed as food similarly and significantly contribute to the maintenance of health and support physiological processes. Additionally, preparations derived from whole mushrooms, due to the more complex profiles of bioactive compounds, have more favorable physiological effects than single extracts (e.g., aqueous-, alcoholic-, or glycerol-based extractions). However, in case of certain fungi species, such as *Penicillium notatum*, only the extract is applied [6,11].

Although the term “medicinal mushroom” is applied mainly to macroscopic fungi such as *Ganoderma lucidum*, *Lentinula edodes* and *Grifolia frondosa* [7], bioactive compounds of microscopic species or other taxonomic categories (i.e., penicillin—*Penicilium notatum*, monacolin K—*Monascus purpureus*) may also play important role in medicine and the development of functional foods [12].

Thus, in this manuscript we use the term “medicinal mushroom” in a broader meaning, which is not related to the taxonomic category, but rather to the meaning of their use in “medicinal application”—since these contain compounds and/or secondary metabolites in high concentrations or in a broad spectrum, which may have positive effects on human health [7,13].

## 2. Nutritional Value of the Main Active Ingredients of Edible and Medicinal Mushrooms

Among the most abundant bioactive compounds of edible and medicinal mushrooms are polysaccharides, particularly β-glucans, which account for 10–50% of the dry matter content [13]. The second most prominent group of compounds are proteins, which typically comprise 10–40% of the dry matter content. Interestingly, many mushroom species contain complete amino acid profiles [4]. The fiber content can reach 20–30%, which beneficial for the gut microbiota [9]. Although mushrooms have relatively low fat content (2–8%), they are typically abundant in unsaturated fatty acids [14]. Triterpenes in mushrooms occur in a lower concentration than polysaccharides or proteins, typically account for 1–5% of the dry matter. Triterpenes are secondary metabolites of fungi with a lanostane-type 30-carbon atoms length structure [15]. In *Ganoderma* species (e.g., *Ganoderma lucidum*, *G. tsugae*) the amounts of these compounds are significantly lower than in other medicinal mushrooms. However, due to their strong anti-inflammatory and cytotoxic effects, these components are important in the physiological effects of these species [16,17]. Moreover, mushrooms provide essential vitamins (e.g., B-complex, D2) and trace elements (e.g., selenium, copper, zinc) in a highly bioavailable form [4,9,16].

## 3. The Ecological Adaptation of Fungi Is Reflected in Their Active Ingredient Composition

Fungi are a diverse group of organisms with a wide range of morphological and ecological characteristics, and their widespread occurrence show the ability to adapt to extreme and unique ecological conditions [18,19]. They may occur on soil, tree bark, decaying wood, forest undergrowth, or on roots either above or below the ground (e.g., *Wolfiporia extensa*, *Tuber macrosporum*). Fungal growth and the accumulation of bioactive compounds are strongly modulated by environmental factors such as light intensity, relative humidity, soil composition, temperature, and the substrate of the growth (substrate: the soil or artificial cultivation media in controlled production of mushrooms).

A striking example of a specialized ecological adaptation is provided by entomopathogenic fungi—i.e., species of the Cordyceps genus—which parasitize insects or other arthropods. These proliferate within their host’s body and grow on the host after its death. Bioactive metabolites produced by *Cordyceps* species, including cordycepin, cordycepic acid, cordymin, β-glucans, and mannoglucans, facilitate the survival of fungi by evading the host’s immune system and efficient nutrient uptake from the host. The biosynthesis of these compounds are the results of highly specialized life cycles and adaptation to host-associated stress conditions [20,21]. The example of the aforementioned fungal genus clearly illustrates how the ecological role and environmental adaptation of fungi are closely linked to the quality of their bioactive compounds and their therapeutic use [22,23].

Numerous studies have demonstrated that individuals of the same fungal species, when cultivated or grown wild under different ecological conditions, may represent significant variations in their polysaccharide, triterpene, or antioxidant content—which influence their physiological effects [4,16]. Nowadays, medicinal mushrooms are primarily cultivated to ensure their standardized bioactive composition—an essential requirement for their targeted application.

## 4. Major Bioactive Compounds of Edible and Medicinal Mushrooms—Chemical Structures, and Their Effects

Several bioactive compounds of edible and medicinal mushrooms have been known and investigated to date (Supplementary S1). Of these, polysaccharides, triterpenes, and nucleosides seem to be the most important in the prevention and treatment of diabetes and cancer.

### 4.1. Polysaccharides/β-Glucans

**Figure 1:**
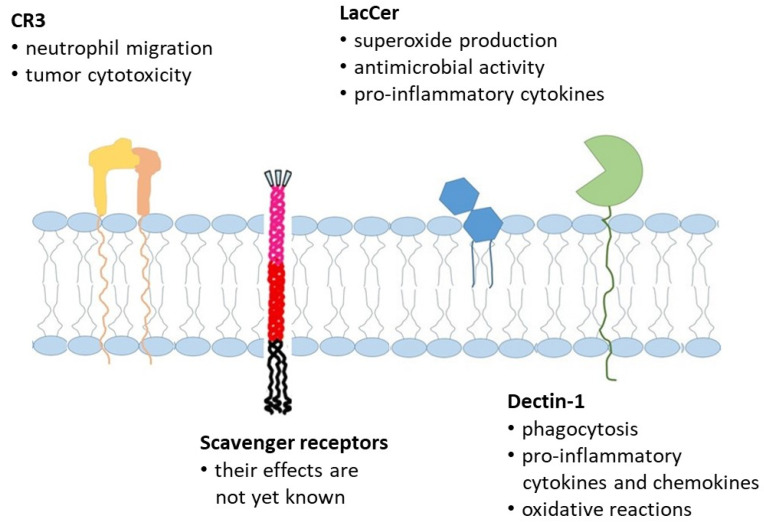
Effects mediated by the pattern recognition receptors (PRRs) of the immune system [30].

The polysaccharide β-glucans are primarily composed of/1 → 3/-β-D-glucose units, which may have side chains attached via/1 → 4/or/1 → 6/cross-linking [24]. In mushrooms, the/1 → 3/- and/1 → 6/- bindings dominate, while in oat and barley the/1 → 3/- and/1 → 4/are mainly presented [25]. Both compositions have important roles in the biological processes, which has been known for a long time [26,27]. Mushroom polysaccharides, particularly β-glucans, are important due to their immunomodulatory and antidiabetic effects [23,28,29]. This aforementioned structure is crucial in the activation of the immune system, whereas other types of glucans do not possess this feature or possess it only to a limited extent. This specific three-dimensional conformation enables these molecules to bind to pattern recognition receptors (PRRs) with high affinity. The β-1,3- and β-1,6-bonded glucans primarily bind to dectin-1, complement receptor 3 (CR3), and toll-like receptor 2/6 (TLR2/6) located on the surface of monocytes, macrophages, dendritic cells, and neutrophil granulocytes (Figure 1) [30,31].

Lentinan, a β-glucan polysaccharide compound derived from *Lentinula edodes*, is a clinically studied immunostimulant that is used in cancer therapy in Japan [32]. β-glucans also be used as complementary therapy in the alleviation of side effects of chemotherapy in cancer patients [13,33].

#### 4.1.1. Immunomodulation

Activation and suppression: β-glucan-type polysaccharides mediate immunomodulation primarily through the activation of innate immune effectors such as natural killer (NK) cells and macrophages [34]. These molecules may decrease the levels of pro-inflammatory cytokines (i.e., TNF-α, IL-6) and inhibit the NFκB signaling pathway [35], but due to infection, they increase these to support the body’s defenses [22]. They modulate the immune response by either stimulation or suppression, depending on the current physiological state of the organism [10,36]. In chronic inflammation β-glucans may have anti-inflammatory effects by attenuating excessive cytokine responses, thus playing a balancing role rather than acting solely as stimulators [37].

Signaling pathway: Dectin-1-mediated signaling is activated through the Syk–CARD9–NF-κB pathway, which modulates the innate immune response [38]. It stimulates the production of cytokines (e.g., IL-6, TNF-α, IL-12) against pathogens—including pathogen fungi—complement activation, enhances phagocytosis, and at the same time may support anti-inflammatory response, preventing septic shock [39].

Immune cell type specific actions: β-glucans stimulate the activity of phagocytic cells, promote antigen presentation, and thereby facilitate the induction of the adaptive immune response [37,40]. CR3-mediated activation enhances the cytotoxic functions of natural killer cells, leading to increased anti-tumor and antiviral activity [40]. β-glucans can induce a Th1/Th17-dominant response by dendritic cell, leading more effective immune response against intracellular pathogens and tumor cells [28].

Gut microbiome modulation: β-glucans have prebiotic effects by promoting the proliferation of health-supporting bacteria (e.g., *Bifidobacterium* spp., *Lactobacillus* spp.), which stimulate the production of epithelial tight junction proteins, thereby reducing intestinal permeability and consequent endotoxemia [41,42].

#### 4.1.2. Antidiabetic Effect

Digestible and non-digestible polysaccharides of mushrooms, improving insulin sensitivity and reducing blood glucose level, may contribute to the management of diabetes [43,44]. The effects of the components of edible and medicinal mushroom in the prevention of insulin resistance and subsequent diabetes have been detailed in our previous review [1]. Here below we just briefly list some findings with references—further details can be found in the original article.

Delayed glucose absorption: β-glucans, acting as soluble dietary fibers, form viscous gels in the intestine, thereby slowing down glucose absorption, attenuating postprandial hyperglycemia, and attenuating insulin response [1,45]. β-glucans and other polysaccharides in certain mushrooms can inhibit the activity of α-glucosidase and α-amylase; this action results in delayed glucose absorption and reduced glycemic response [46,47].

Improved insulin sensitivity: β-glucans reduce TNF-α and IL-6 cytokine levels, increase the fasting serum insulin level, reactivate the insulin signaling, and increase AMPK enzyme activity, which plays a pivotal role in glucose homeostasis [48,49,50,51]. Their anti-inflammatory effects may attenuate chronic low-grade inflammation, which represents one of the main causes of insulin resistance [34,35,36].

β-cells protection and regeneration: β-glucans derived from *Lentinula edodes*, *Ganoderma lucidum*, and *Grifola frondosa* have been shown to reduce oxidative stress in the pancreatic islets of Langerhans and protect these cells from apoptosis, while also supporting their insulin secretion [44].

### 4.2. Triterpenes

Triterpenes possess a lanostane-type structure (Supplementary S2). They have multiple functional groups, which contribute to their effects [15,52]. Due to their lipophilic nature, these triterpenes are often extracted by specific and uncommon methods, which preserves the bioactive forms of these compounds [53,54].

Triterpenoids, especially from *Ganoderma lucidum*, had been shown to possess anti-inflammatory, hepatoprotective and anticancer effects [16]. Through their anti-inflammatory, antioxidant properties, the inhibition of enzymes and production of advanced glycation end products, through the enhancement of GLUT-4, GLP-1, adiponectin, insulin receptor substrate (IRS) and the PI3K/Akt signaling pathway expression, glucose uptake, glycogen synthesis, and thus insulin sensitivity, and triterpenes support metabolic homeostasis in a complex way [55,56]. Its cytotoxic and apoptotic ability in human cancer cell lines (including hepatic, colorectal, and breast carcinomas) are described, which are mediated partly through the activation of mitochondrial caspases [17].

The trametenolic acid isolated—among others—from *Inonotus obliquus* has anti-inflammatory and anti-tumor capacity [57,58]. It protects kidney function by reducing inflammation and oxidative stress via the Nrf2/HO-1 and NF-κB signaling pathways [59]. Ganoderic acids and lucidenic acids from *Ganoderma* species may contribute to the regulation of glucose metabolism as well [60]. Several triterpenoids isolated from *Wolfiporia cocos* have been shown to enhance glucose uptake and reduce blood glucose levels [61]. In preclinical studies, diabetic-model animals treated with the triterpene fraction of Antrodia cinnamomea significantly reduced blood glucose levels, and enhanced insulin signaling and sensitivity were detected [56]. *Hericium erinaceus* contains the neurotropic compounds hericenones and erinacines, of which erinacines are triterpenoids. These promote neuronal regeneration and are therefore considered promising in the complementary treatment of neurodegenerative disorders such as Alzheimer’s disease (AD) [62]. Recent findings suggested that AD may represent a brain-specific form of diabetes (type 3 diabetes) [63].

**Figure 2:**
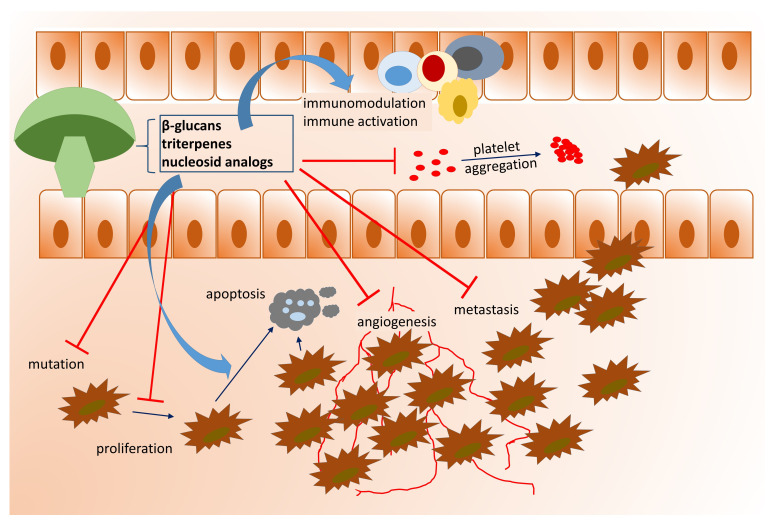
Anti-tumor effects of triterpenes, β-glucans and nucleoside analogs isolated from medicinal mushrooms [17,22,64,65,66]. Blue arrow: activating, Red arrow with line head: inhibiting, Black arrow: showing a process.

Triterpenes, together with β-glucans and nucleoside analogs, have significant anti-tumor effects (Figure 2) [17,22,64,65,66].

### 4.3. Nucleoside Analogs (Nucleic Acid Base-, Nucleoside-, or Nucleotide-Variants)

Nucleoside analogs are widely used as antimicrobial, anti-tumor, cardioprotective agents because of their immunomodulatory properties and positive effects on cardiovasculature [23,36]. They have long been known, and are, approved and applied drugs in cancer treatments [67,68]. Nucleosides and their analogs found in medicinal mushrooms (i.e., adenosine, cordycepin, clitocine, inosine—cordycepin and clitocine are adenosine analogs; Supplementary S3) originated from the catabolism of nucleic acids. However, similar to those originated in another way or from other sources, these also show a wide variety of pharmacological effects, which support health, disease prevention and treatments [68,69,70]. Cordycepin (3′-dezoxyadenosine) has anticancer potential and has been shown to enhance microcirculation—thus improving tissue perfusion and protecting myocardium [71].

#### 4.3.1. Antimicrobial Effects

Cordycepin showed moderate anti-HCV effects and augmented the effect of interferon and ribavirin therapy in HCV RNA-producing cells [72]. Moreover, clitocine from the edible mushroom *Clitocybe inversa*—similar to those of bacterial origin, i.e., oxetanocin A (*Bacillus megaterium*), spicamycin (*Streptomyces alanosinicus*) and anicemycin (*Streptomyces thermoviolaceus*)—also have antiviral, and additionally, antibacterial effects [69].

#### 4.3.2. Anti-Tumor Effects

The multi-compound or complete extracts of *Cordyceps* spp. demonstrated anti-tumor effects on several cell lines and in vivo tumor models [73]. It inhibits the synthesis of RNA [72], and may shut down translation through AMPK activation, which inhibits mTOR signaling [71]. Moreover, it induces both the extrinsic and intrinsic apoptotic pathways and inhibits the proliferation, growth and the development of metastasis [71,72]. The effects of adenosine on tumor growth and on the tumor microenvironment are complex: they are tumor- and immune cell-dependent. They can play both stimulatory and inhibitory roles depending on the cell type and the different adenosine receptors [74]. Clitocine, similarly, has significant anti-tumor effects [69].

#### 4.3.3. Cardioprotective, Cardiovascular Effects

Adenosine is an effective agent in chronic heart failure [70,75]. Cordycepin inhibits collagen-induced platelet aggregation via reduction in Ca^2+^ and thromboxane A2, and elevation of intracellular cAMP and cGMP level [71]. Nucleosides and nucleobases in *Ganoderma lucidum* also can inhibit platelet aggregation and reduce the elevated serum aldolase levels [70].

#### 4.3.4. Immunomodulatory Effects

Adenosine and cordycepin can activate NK cells and the phagocytic function of macrophages [22]. They affect almost all the cell types of the immune system and also have significant impacts on cytokine production [73].

## 5. β-Glucans, Triterpenes and Nucleoside Analogs as Complementary Therapy in Type 2 Diabetes and Cancer—Summary of the Results Reported in Clinical Trials

In this review we collected the accessible clinical trials/human studies that investigated mushroom components β-glucans, triterpenes, and nucleoside analogs as complementary therapies in diabetes and four cancer types (breast, prostate, lung and colorectal cancers) from the scientific database of PubMed. Searching terms were: “(cancers) AND (clinical trials) AND (medicinal mushrooms)”, “(cancers) AND (clinical trials) AND (beta glucans)”, and “(diabetes) AND (clinical trials) AND (mushrooms)”. Of the 254 articles 13 were related to breast cancers, four to prostate cancers, nine to lung cancers, and 18 to colorectal cancers, and nine were related to T2DM.

In relation to impaired glucose tolerance (IGT) or type 2 diabetes (T2DM), nine manuscripts that investigated the effects of edible or medicinal mushrooms were found (Supplementary S4). Three of them applied mushroom extracts (high molecular weight polysaccharide fractions which are mainly glucans/β-glucans, or low molecular weight extracts which are primarily terpenoids, polyphenols and small peptides) and the others applied mushroom powders as complementary therapy.

Patients with IGT had significantly increased GLP-1 and a significantly decreased non-esterified free fatty acids (NEFAs) amount after a *Pleurotus ostreatus*-enriched meal in a randomized controlled crossover study [45]. Most of the study found significantly improved metabolic parameters in T2DM patients such as decreased fasting and/or postprandial glucose levels and increased postprandial insulin levels [76,77], significantly decreased HbA1c levels [77,78], significantly improved lipid profile [78,79,80], significantly increased adiponectin levels, significantly decreased homeostatic model assessment of insulin resistance (HOMA-IR) index [81,82], significantly increased oxygen radical absorbance capacity and significantly decreased advanced glycation end products (AGEs) [82]. A double-blinded, randomized controlled study found significantly improved quality of life (QoL) in T2DM patients who consumed oyster mushroom biscuits together with diet and exercise as complementary therapies [78]. However, one article, a systematic review and meta-analysis of randomized controlled clinical trials, has not found significant changes either in plasma glucose, nor in blood pressure or triglycerides [83]. The difference may have resulted from that the clinical trials in this systematic review applied 1.4–3 g mushroom powder per day, while all the others mentioned above used mushroom or mushroom extracts per day in a higher amount (9.2–100 g/day)—except one (with 1.5 g/day but this was an extract). One study investigated the safety of the polysaccharide extract of *Ganoderma lucidum* (Ganopoly) and found that it was well tolerated [77].

In human studies and/or clinical trials discussing the effects of edible or medicinal mushroom powder or extracts on the four main cancer types we found 13 articles for breast, four for prostate, nine for lung cancers, and 18 for colorectal cancers which had at least accessible abstracts (Table 1, Table 2 and Table 3).

Of the 13 breast cancer studies (Table 1), three examined and found significantly improved QoL, and two of these found decreased symptoms after mushroom/or extract intake [84,85,86]. One of the 13 papers studying the safety of *Lentinula edodes* mycelia extract (L.E.M.) found no adverse events attributable to L.E.M. [85]. Similarly, lentinan (LNT, β-glucans extracted from *Lentinula edodes*) injection has been shown to be safe as complementary therapy after surgery [87]. Another study, a phase I/II trial found that Maitake polysaccharide extract over a 3-week period was well tolerated and no dose-limiting toxicity was seen up to 10 mg/kg per day [88].

Six studies investigated the immunomodulatory effects of β-glucan or polysaccharide extracts [85,87,88,89,90,91]. Two of them found significantly increased number of certain immune cells, i.e., monocytes [90], T helper and B cells [91] after the complementary treatments. Two found significantly increased activation of immune system, i.e., significantly increased NK cell activity with significantly decreased level of immunosuppressive acidic protein (IAP) [85], and significantly increased CD95 (death-receptor, mediator of phagocytosis-induced cell death) and CD45RA (an indicator of cellular activation) expressions on monocytes [90]. A double-blind placebo controlled randomized clinical trial reported significantly increased serum level of IL-4 in intervention vs. placebo group. Additionally, significant and opposite change was observed in the serum level of IL-12 between the two groups: IL-12 increased in the intervention group while it decreased in the placebo group after intervention [89]. Another clinical trial recorded significantly decreased levels of plasma soluble IL-2 receptor (sIL-2R), which is a clinical marker of immune system activation [91]. Additionally, LNT injection after adrenalectomy and oophorectomy (Ax + Ox) in patients with recurrence breast cancer induced tumor atrophy and intense infiltration of T, B cells and macrophages into stroma around the tumor [87]. Although the results described above suggest a clear immune system-activating effect of mushroom extracts, a phase I/II dose-escalation clinical trial suggested a non-linear, more hyperbolic relationship for most immunological variables. It means not a maximum dose but more an optimal dose may exist which varies for different immunological parameters—thus the same dose may activate certain parameters while inhibiting others [88]. The authors suggest that because these natural components produce more complex effects than expected, cancer patients should be made aware of the fact that these can both enhance and suppress the immune system [88].

Five studies investigated the effects of β-glucan or polysaccharide extracts on survival in breast cancer patients. One of them found significant life span prolongation in LNT groups vs. the control after application of LNT as supportive therapy [92]. A randomized clinical trial (RCT) reported significantly increased 5-year relapse-free survival in ER+ patients treated with a combination therapy of tamoxifen (TAM) with polysaccharide-K, krestin (PSK), compared to the TAM-only group. In this study, the survival of ER- patients was not improved by immunomodulatory adjuvant therapy of PSK [93]. However, in another RCT where a subgroup analysis was applied, significantly increased overall survival was found in node-negative, ER-negative Stage IIA (T2N1) breast cancer patients after combination therapy with PSK [94]. Similar to the aforementioned two studies, generally, without subgroup analysis, there was no difference in survival between the FEMP (5-FU, cyclophosphamide, mitomycin C, prednisolone) and FEMP + PSK groups in a randomized controlled clinical trial of breast cancer patients [95]. However, in a more stratified clinical trial, breast cancer patients with the presence of HLA antigen B40+ had significantly better 5- and 10-year disease-free survival (DFS) than B40− patients in the FEMP + PSK group. Additionally, there was no significant difference in DFS between B40+ vs. B40- in the FEMP-only group [96].

**Table 1:** Clinical trials applying edible/medicinal mushrooms or their extracts at breast and prostate cancer patients. Randomized clinical trial (RCT), quality of life (QoL), global health status (GHS), immunosuppressive acidic protein (IAP), white blood cell (WBC), lentinan (LNT), adrenalectomy + oophorectomy (Ax + Ox), polysaccharide-K or Krestin (PSK), mitomycin C (MMC), disease-free survival (DFS), overall survival (OS), prostate specific antigen (PSA).

Disease Type	Complementary Treatment and/or Treatment(s)	Outcomes	Compared Groups	Key Findings	N all (Group1/Group2/etc.)	Study Type	Ref
breast cancer	Two 10 mg capsules of soluble 1–3, 1–6, D-beta-glucan daily 21 days (between two courses of chemotherapy)	Global health status (GHS)/ quality of life (QoL)	treatment/placebo	GHS/QoL no change but symptom score significantly decreased (*p* = 0.048) in treatment vs. placebo.	30 (15/15)	double-blind placebo controlled RCT	[84]
breast cancer (+gastrointestinal cancer)	*Lentinula edodes* mycelia extract (L.E.M.)	Safety, quality of life (QoL) score, immune system activation	pre-chemo state vs. a, first course of chemo alone and b, second course of chemo + L.E.M	L.E.M is safe. a, No significant change. b, Improved QoL (*p* < 0.05), NK cell activity (*p* < 0.05), and reduced IAP levels (*p* < 0.01).	3 (+2+2)	pilot study	[85]
breast cancer	*A. sylvaticus* (2.1 g/day)	Nutritional status, side effects	*A. sylvaticus* group/placebo group	Six chemo cycle group (6×): improved nutritional status in A.s. group vs. placebo, reduced side effects	46 (23/23); 6×: 10/10	randomized, placebo-controlled, double-blind, clinical trial	[86]
breast cancer	2 × 10 mg capsules of soluble 1–3, 1–6, D-beta-glucan daily 21 days (between two courses of chemotherapy)	Serum IL-4 and IL-12	treatment/placebo	Intervention vs. placebo: decreased IL-4 (*p* = 0.001), increased IL-12 (*p* = 0.007)	30 (15/15)	double- blind placebo controlled RCT	[89]
breast cancer	Oral 1–3, 1–6, D-beta-glucan daily	Peripheral blood monocytes	treatment/control	Increased: monocyte count (*p* = 0.015), CD95 expression (*p* = 0.002), and CD45RA expression (*p* = 0.001).	39 (23/16)	clinical trial	[90]
breast cancer	Daily for 6 months: Yunzhi (*Coriolus versicolor*) 50 mg/kg body weight, 100% polysaccharopeptide (PSP) and Danshen (*Salvia miltiorrhiza*) 20 mg/kg body weight.	Post-treatment immunological function	2nd, 4th, 6th months vs. starting point	Significant increases CD4+ T-helper lymphocyte absolute count (*p* < 0.05), CD4+/CD8+ ratio (*p* < 0.05) and B-lymphocyte % and absolute count (*p* < 0.05). Significant decrease in plasma soluble IL-2 receptor (sIL-2R) level (*p* < 0.05)	82	clinical trial	[91]
breast cancer	Maitake polysaccharide extract was taken orally at 0.1, 0.5, 1.5, 3, or 5 mg/kg twice daily for 3 weeks	Safety and toxicity, immunological effects	treatment compared to baseline	Well tolerated. No dose-limiting toxicity up to 10 mg/kg per day. Complex effects: depress or enhance the different immune functions.	34	phase I/II dose-escalation clinical trial	[88]
breast cancer (recurrence)	Lentinan (LNT) injection	Efficacy and safety of LNT post-treatment		LNT injection following surgical therapy (Ax + Ox): a, greater regression in surgery alone, b, LNT-surgical therapy resulted tumor atrophy, intense infiltration of T cells, B cells and macrophages into stroma around tumor. LNT is effective and safe.	33	clinical studies	[87]
breast cancer	Lentinan (LNT) as supportive therapy	Survival	treatment/control	Significant life span prolongation in LNT groups vs. control (*p* < 0.05).		RCT with envelope method	[92]
breast cancer	Postoperative therapy—mitomycin C (MMC) in ER+ and ER- stage II patients. ER+: TAM or TAM + FT or TAM + PSK; ER-: FT or PSK (3 g/day)	Survival	ER+: **A** (TAM); **B** (TAM + FT); **C** (TAM + PSK) ER-: **D**—FT; **E**—3 g/day PSK	ER+ patients: 5-year relapse-free survival in B vs. A and C (*p* = 0.010). ER− patients: No significant difference in 5-year OS or RFS between D and E.	ER+ (525)/ ER- (376)	randomized clinical trial	[93]
breast cancer	Polysaccharide-K or Krestin (PSK) 3 g/day	Survival	TAM + chemo/TAM + chemo + PSK	No overall and relapse-free survival (OS and RFS) difference with or without PSK in ER-negative patients. Subgroup analysis: PSK benefit in OS in node-negative, ER-negative Stage IIA (T2N1) (*p* = 0.0017).	914	randomized clinical trial	[94]
breast cancer	FEMP (5-FU, cyclophosphamide, mitomycin C, prednisolone) /FEMP + LMS (levamisole)/ FEMP + PSK (polysaccharide K)	Disease-free survival (DFS) overall survival (OS) safety	FEMP/FEMP + LMS/FEMP + PSK	No difference in DFS or OS among the three groups. Adverse events: low incidence, mild, well tolerated	227	randomized controlled clinical trial	[95]
breast cancer	No adjuvant therapy/ FEMP (5-FU, cyclophosphamide, MMC, prednisolone)/FEMP + PSK (3 g/day) for 28 day (two course in a year for 5 years)	Disease-free survival (DFS) in B40+ and B40- patients	no adjuvant therapy/FEMP/FEMP + PSK	FEMP-only group: No difference in DFS in between B40+ vs. B40-. FEMP + PSK group: significantly better DFS in B40+ patients (100%) than in B40- patients (76%, 55%).	134	retrospective randomized controlled clinical trial	[96]
prostate cancer	White button mushroom (WBM) powder 4, 6, 8, 10, 12 or 14 g/day	Safety, PSA response, immunological effects	baseline vs. post-treatment	Safety: no dose-limiting toxicities. PSA Response: overall PSA response rate: 11%, 2 complete, 2 partial responses after 3 months. Responders: higher baseline IL-15 levels, treatment-associated decline in MDSCs.	36	phase I, open-labeled, non-randomized, dose-escalation clinical trial	[97]
prostate cancer	Mushroom mycelium extract (AHCC) 4.5 g/day for 6 months	PSA response	before/ after 6 months	No significant PSA change pre- vs. post-treatment.	74	phase II, open-label, single-arm clinical trial	[98]
prostate cancer	SME (shiitake mushroom extract) three times daily for 6 months	PSA response	PSA progression rate before/during SME treatment	By 6 months: 23 patients experienced disease progression 38 patients had stable PSA levels	61	open-label clinical study	[99]
prostate cancer	Hormone and chemotherapy (FT 400–800 mg/day) + lentinan (2 mg/week inpatients; 4 mg/2 weeks outpatients) for at least 3 months	Survival	treated/control	5-year survival 43% vs. 29% (*p* < 0.05) lentinan vs. control	69 (33/36)	prospective randomized multicenter study	[100]

Four studies investigated the effects of edible or medicinal mushroom powder or extracts on prostate cancers (Table 1) [97,98,99,100]. A phase I, open-labeled, non-randomized, dose-escalation clinical trial investigated safety and found no dose-limiting toxicity of white button mushroom powder [97]. Three studies evaluated PSA changes, and only one reported stabile prostate specific antigen (PSA) levels in >50% of patients [99]—the others found worse results [97,98]. One studying immunological functions showed significantly higher baseline IL-15 levels in responders and significantly decreased level of MDSCs (CD33+HLA-DR) at week 13 compared to the time of enrolment of prostate cancer patients [97]. One study found significantly increased 5-year survival in the lentinan group vs. the control in prostate cancer patients [100].

One of the nine lung cancer articles (Table 2), a meta-analysis (of 17 randomized clinical trials) reported significantly improved QoL in a combination therapy with lentinan (RR = 1.48 (95% CI: 1.27–1.72; *p* < 0.00001) compared to chemotherapy alone [101]. This meta-analysis found lower incidence and degree of adverse reactions in combination therapy than in cisplatin thoracic injection alone. Similarly, in a double-blind, placebo-controlled, randomized study, significantly less patients were withdrawn from the study because of disease progression in PSP group than in placebo (5.9 and 23.5%, respectively; *p* = 0.04; OR 4.00) [102]. BTH1677, an 1,3–1,6 beta-glucan extract from *Saccharomyces cerevisiae*, was well tolerated in a randomized, open-labeled, multicenter, phase II clinical trial [103], similarly to another beta-glucan product, which was used in a self-initiated, single-arm, phase II clinical trial [104]. An open-label clinical study investigating immune function found marked interindividual variability and no significant overall changes [105]. While in a double-blind, placebo-controlled, randomized study, PSP significantly increased IgG and IgM (*p* = 0.02 and 0.04) [102]. The efficacy of combination therapy with lentinan was significantly higher (RR = 1.34 (95% CI: 1.21–1.48; *p* < 0.0007) than cisplatin alone, as calculated by the meta-analysis [101]. BTH1677 significantly increased overall response rate vs. the control (ORR investigator: 47.8% vs. 23.1%, *p* = 0.0468; however, the ORR central: 36.6% vs. 23.1%, *p* = 0.2895). Additionally, the presence of anti-beta-glucan antibody (ABA) significantly increased the median overall survival (OS) in lung cancer patients (OS ABA- vs. ABA+: HR 0.48 [95% CI: 0.24, 0.95]; *p* = 0.0327) [103]. It was reported that beta-glucan can reverse resistance to anti-PD-1/PD-L1 inhibitors [106], therefore in a single arm phase II clinical trial, non-small cell lung cancer patients who have previously failed anti-PD-1 therapy received beta-glucan, Envafolimab and Endostar. The overall response rate (ORR) was 21.7%, the median overall survival was 9.8 months (95% CI: 7.2–12.4), the median progression-free survival (mPFS) was 4.3 months (95% CI: 2.0–6.6), and mPFS of PD-L1+ vs. PD-L1- patients were 6.3 vs. 2.3 months (*p* = 0.002). Favorable response was associated with increased CD40-L and EGF levels while resistance was associated with increased CASP-8, ARG1, MMP12, CD28, and CXCL5 levels [104]. Three other studies similarly found significantly increased survival rates after combination therapy with PSK compared to controls [107,108,109]. Moreover, a pooled analysis of 38 clinical trials reported significantly increased ORR lentinan + chemotherapy vs. chemotherapy alone (56.9% vs. 43.3%; *p* < 0.001), where the pooled RR was 0.79 (95% CI: 0.74–0.85) [32].

**Table 2:** Clinical trials applying edible/medicinal mushrooms or their extracts at lung cancer patients. Randomized clinical trial (RCT), risk ratio (RR), overall response rate (ORR), disease control rate (DCR), median progression-free survival (mPFS), overall survival (OS), polysaccharide peptide (PSP), anti-beta-glucan antibody (ABA), median survival time (MST), polysaccharide-K or Krestin (PSK).

Disease Type	Complementary Treatment and/or Treatment(s)	Outcomes	Compared Groups	Key Findings	N all (Group1/Group2/etc.)	Study Type	Ref
lung cancer	Lentinan combined with cisplatin thoracic injection	effectiveness, quality of life (QoL), safety	treated/ control	Effectiveness: combination therapy vs. cisplatin alone: RR = 1.34 (95% CI: 1.21–1.48; *p* < 0.0007). QoL improved with combination therapy RR = 1.48 (95% CI: 1.27–1.72; *p* < 0.00001) Adverse events lower in combination therapy	1390 (711/679)	meta-analysis (17 randomized clinical trials)	[101]
lung cancer	Purified Yunzhi PSP capsules (340 mg each)/placebo (350 mg sucrose each)/ three times daily	morbidity	PSP (polysaccharide-peptide)/ placebo for 28 days	Significantly less disease progression PSP vs. control (5.9 and 23.5%, respectively; *p* = 0.04; OR 4.00). PSP significantly increased IgG and IgM (*p* = 0.02 and 0.04), and body fat% (*p* = 0.08).	68 (32/26)	double-blind, placebo-controlled, randomized study	[102]
lung cancer	BTH1677 (1,3–1,6 beta glucan, 4 mg/kg weekly) with chemotherapy (cetuximab/carboplatin/paclitaxel)	tolerability and efficacy	treatment/control	BTH1677 well tolerated. BTH1677 increased ORR (investigator: 47.8% vs. 23.1%, *p* = 0.0468; central: 36.6% vs. 23.1%, *p* = 0.2895). median OS ABA-vs ABA+: HR 0.48 [95% CI: 0.24, 0.95]; *p* = 0.0327)	90 (59/29)	randomized open-labeled, multicentre, Phase II CT	[103]
lung cancer	β-glucan (500 mg, b.i.d, d1–21), Envafolimab (300 mg, d1) and Endostar (210 mg, c.i.v72h) in every 3 weeks	clinical efficacy and safety		ORR: 21.7%, DCR: 73.9% mPFS: 4.3 months (95% CI: 2.0–6.6) mOS: 9.8 months (95% CI: 7.2–12.4) mPFS (PD-L1+ vs. PD-L1): 6.3 vs. 2.3 months (*p* = 0.002) adverse events: (AEs) 52.2% (Gr 3 AEs: 8.7%) Treatment resistance: increased CASP-8, ARG1, MMP12, CD28, CXCL5. Favorable response: increased CD40-L, EGF.	23	self-initiated single-arm, Phase II CT	[104]
lung cancer	5.4 g/day Ganopoly (*Ganoderma lucidum* polysaccharides) for 12 weeks	immune function	comparison within-subject: before/after treatment Ganopoly	No significant changes. Marked interindividual variability. Subgroup of patients showed notable immune modulation.	30	open-label clinical study	[105]
lung cancer	Intravenous injection of lentinan (1–1.5 mg/day)	efficacy	treated/ control	ORR increased: 43.3% to 56.9% (*p* < 0.001) chemotherapy alone vs. lentinan + chemotherapy; Pooled efficacy: RR = 0.79 (95% CI: 0.74–0.85, I^2^ = 11%)	3117 (1592/1525)	pooled analysis of clinical trial (38 clinical trials)	[32]
lung cancer	Polysaccharide-K or Krestin (PSK)	efficacy	radiation therapy (RT) + PSK/RT	5-year survival rates in stage I–II and stage III: RT + PSK 39% and 26% vs. RT-only 17% and 8%, respectively. PSK significantly improved survival in RT responders.	170	retrospective study	[107]
lung cancer	PSK 3 g/day	response rate, survival time	chemotherapy/immuno-chemotherapy + PSK	Stage III subgroup: RR: 11.1% and 37.5% chemotherapy vs. immunochemotherapy + PKS (*p* = 0.046) median survival time (MST): 457 days vs. 576 days (*p* = 0.075)	138	randomized study	[108]
lung cancer	PSK and OK-432 (Picibanil) as biological response modifiers (BRMs)	survival	chemotherapy + PSK/chemothe-rapy + OK-432/chemothe-rapy alone	4-year survival rates: control 34.7%, OK-432 36.7%, PSK 55.9%. Survival rates for stage III: significant immunochemotherapy vs. chemotherapy alone	113	randomized clinical studies	[109]

Of the 18 studies investigating the effects of mushroom or mushroom extracts in colorectal cancer patients (Table 3), one studied the effects on QoL and showed improvement after a complementary treatment with *Agaricus sylvaticus* fungus (30 mg/kg/day) for 6 months [110]. One investigating the safety of beta-glucan found no grade 4 toxicity, no treatment-related deaths, and adverse event-related patient discontinuation [111].

Two articles examined changes in metabolic parameters; both were based on the same randomized double-blind, placebo-controlled clinical study of *Agaricus sylvaticus* fungus (30 mg/kg/day) for 6 months [112,113]. The first found significantly decreased plasma glucose (*p* = 0.02), total cholesterol (*p* = 0.01), creatinine (*p* = 0.05), aspartate aminotransferase (*p* = 0.05), alanine aminotransferase (*p* = 0.04), IgA (*p* = 0.0001), IgM (*p* = 0.02), systolic blood pressure (*p* = 0.0001) and diastolic blood pressure (*p* = 0.0001) [112]. Similarly, the second recorded significantly reduced fasting plasma glucose level in the *Agaricus sylvaticus* group after six months (*p* = 0.01), while significantly increased levels were found in the placebo group after both three and six months (*p* = 0.03 and *p* = 0.01, respectively) [113].

Four studies investigated hematological and immune parameters [114,115,116,117]. The randomized double-blind, placebo-controlled clinical study of *Agaricus sylvaticus* fungus (30 mg/kg/day) showed significant increase in hemoglobin (*p* = 0.0001), hematocrit (*p* = 0.0001), erythrocytes (*p* = 0.01), mean cell volume (*p* = 0.01), mean cell hemoglobin (*p* = 0.0001), mean cell hemoglobin concentration (*p* = 0.0001), neutrophil levels (*p* = 0.0001), and significant decrease in platelet count (*p* = 0.03)—which remained within normal levels—in the *A. sylvaticus* group after treatment for 6 months [114]. In the randomized controlled study of the intake of 3.0 g PSK orally before surgery for the short-term (<14 days), significantly increased peripheral blood lymphocyte (PBL) and significantly decreased CD9+11b+ suppressor T cells in regional node lymphocyte (RNL) in pre- vs. post-comparison. Moreover, applying for long-term (≥14 days) PSK significantly increased cytotoxicity against myelogenous leukemia and gastric cell lines, and the levels of CD16+ cell proportion and CD4+Leu8− helper T cells in RNL [114]. According to the authors, the immunological effects of PKS depend on the duration, not its frequency of administration, and are suggestive of modulation of suppressor cells in regional lymph nodes [114]. A clinical trial found significant reduction in serum sIL-2R (*p* < 0.05) and IL-10 production after 2 months of PSK complementary treatment with chemotherapy (*p* < 0.05) [117]. In contrast, a non-randomized open-labeled clinical trial applying *Ganoderma lucidum* (5.4 g/day) orally for 12 weeks, with equal or more than 4 weeks of interval between prior chemotherapy or radiotherapy, found no statistically significant differences between the pre- vs. post-treatment values of immune functions [115].

A total of 11 clinical trials have been conducted to investigate the efficacy and survival rates of edible/medicinal mushrooms or their extracts in patients with colon cancer. Two of these found significant life span prolongation after lentinan (LNT) treatment in combination with chemotherapy (*p* < 0.01 and *p* < 0.05) [92,118]. Five of the eight trials applying PKS in combination with chemotherapy found significantly increased survivals (DFR and/or OS) in PKS group vs. placebo/chemotherapy alone either in the whole or in the subgroup of enrolled patients [119,120,121,122,123]. The other three found no significant differences between the compared groups, similarly to that applied imprime PGG [111,124,125,126].

**Table 3:** Clinical trials applying edible/medicinal mushrooms or their extracts at colorectal cancer patients. Randomized clinical trial (RCT), quality of life (QoL), overall response rate (ORR), polysaccharide-K or Krestin (PSK), lentinan (LNT), overall survival (OS), disease-free survival (DFS).

Disease Type	Complementary Treatment and/or Treatment(s)	Outcomes	Compared Groups	Key Findings	N all (Group1/Group2/etc.)	Study Type	Ref
colorectal cancer	*Agaricus sylvaticus* fungus (30 mg/kg/day) for 6 months	quality of life (QoL)	Agaricus sylvaticus/placebo (baseline vs. after treatment)	*A. sylvaticus* group: **improved:** adhesion to physical activity; mood, sleep quality, appetite, **reduced**: constipation, diarrhea, postprandial fullness, nausea, abdominal distention and pain	56 (28/28)	randomizeddouble-blind, placebo-controlled clinical study	[110]
		hematological and immune parameters		After treatment in *A. sylvaticus* group: **increased:** hemoglobin (*p* = 0.0001), haematocrit (*p* = 0.0001), erythrocytes (*p* = 0.01), neutrophil levels (*p* = 0.0001). **decreased:** platelet count (*p* = 0.03) within normal range			[114]
		metabolic parameters and blood pressure		Reduction after treatment in *A. sylvaticus* group in: plasma glucose (*p* = 0.02), total cholesterol (*p* = 0.01), creatinine (*p* = 0.05), aspartate aminotransferase (*p* = 0.05), alanine aminotransferase (*p* = 0.04), IgA (*p* = 0.0001), IgM (*p* = 0.02), systolic blood pressure (*p* = 0.0001) and diastolic blood pressure (*p* = 0.0001).			[112]
		fasting plasma glucose (in post-surgery patients)		**Placebo:** increased fasting plasma glucose after 3 months (*p* = 0.03), and six months (*p* = 0.01). ***A. s.*** **group:** decreased fasting plasma glucose level after six months (*p* = 0.01).			[113]
colorectal cancer	*Ganoderma* *lucidum* 5.4 g/day orally for 12 weeks	immune function	Pre- vs. post-treatment	No significant pre– and post-treatment differences.	41	non- randomized open clinical trial	[115]
colorectal cancer	3.0 g polysaccharide PSK orally before surgery daily or every other day for less, equal or longer than 14 days (cut-off time)	peripheral blood lymphocyte (PBL), regional node lymphocyte (RNL), cytotoxicity	Control/PSK before surgery	**Short-term PSK (<14 days):** significantly increased PBL response and decreased CD9+11b+ suppressor T cells in RN pre- vs. post-treatment. **Long-term PSK (≥14 days):** significantly increased cytotoxicity against K562, KATO-3 cells, CD16+ cell proportion and CD4+Leu8− helper T cells in RNL.	18	randomized controlled study	[116]
colorectal cancer	Cisplatin (twice a week) + UTF (400 mg/day) + polysaccharide K (PSK, 3 g/day) for 2 months	immune regulatory markers	Cisplatin + UFT + PSK compared to baseline	Reduced: serum sIL-2R (*p* < 0.05), IL-10 production after 2 months (*p* < 0.05).	10	clinical trial	[117]
colorectal cancer	Imprime PGG (soluble, beta-1,3/1,6 glucan) weekly intravenously at 4 mg/kg followed by cetuximab	efficacy and safety		**Efficacy**: ORR 5.6% (1 partial response/18 patients) **Safety**: No Grade 4 toxicity. No treatment-related deaths. No adverse event-related patient discontinuation. Study discontinuation because of radiographic disease progression (n = 14, 77.8%).	18	phase II open-label multicentre clinical study	[111]
colorectal cancer	Lentinan (LNT) 1 mg/person/day twice a week or 2 mg/person/day once a week intravenously in combination with mitomycin C (MMC) + 5-FU or tegafur	survival	Treatment/control	Significant life span prolongation in LNT groups vs. control (*p* < 0.01).		randomized controlled clinical studies with envelope method	[92]
colorectal cancer	MMC + 5-FU + lentinan (LNT)	survival	MMC + 5-FU (control) vs. MMC + 5-FU + LNT (LNT group)	Significantly increase in survival rates in LNT group vs. control (*p* < 0.05)	51	randomized clinical study	[118]
colorectal cancer	3 g PSK + 300 mg UFT or 300 mg UFT alone orally each day for 2-year period following i.v MMC	5-year DFS/OS, recurrence, safety	PSK + UFT/UFT	**5-year DFS:** PSK + UFT vs. UFT alone 73.0% vs. 58.8% *p* = 0.016 **5-year OS**: PSK + UFT vs. UFT alone 81.8% vs. 72.1%, *p* = 0.056 **Stage III subgroup:** **DFS**: 60.0% vs. 32.1% (*p* = 0.002); **OS**: 74.6% vs. 46.4% (*p* = 0.003) **Recurrence**: 43.6% reduction, **Lung metastases** reduced OR = 0.27 (95% CI 0.09–0.77) *p* = 0.02 **Recurrence predictors:** regional metastasis (RR 2.973; *p* < 0.001); omission of PSK (RR 2.106; *p* = 0.007); Higher primary tumor (RR 4.398; *p* = 0.047) **Safety**: mild adverse effects, good compliance with UFT and PSK	205 (137/68)	randomized controlled study	[119]
colorectal cancer	5-FU (2 × 24 h/week for 3–4 weeks + PSK (300 mg/day for 4 weeks)— (5-FU and PSK treatments alternated and ten course of alternate administration were conducted)	7-year disease-free survival (DFS), overall survival (OS), cancer-death survival (CDS), recurrence rate	5-FU/ 5-FU + PSK	**Preoperative CEA level ≥ 3.0 ng/mL:** 7-year DFS, OS and CDS better in PSK group vs. control (*p* = 0.019, 0.007, 0.014). **Preoperative purified protein derivative (PPD) level < 19.0 mm:** 7-year DFS, OS, CDS better in PSK group vs. control (*p* = 0.018, 0.036, 0.028). Recurrence significantly less in PSK group.	87 (44/43)	subanalysis of a randomized clinical study	[120]
colorectal cancer	Polysaccharide K (PSK)	disease-free survival (DFS), overall survival (OS), leukocyte (PMN) functional activity	PSK/placebo group	DFS: significantly higher in the PSK vs. placebo (*p* < 0.05). OS: significantly higher in PSK vs. control (*p* < 0.05). Immunologic findings: enhanced locomotion and phagocytic activity of PMNs from PSK vs. placebo	111 (56/55)	randomized double-blind trial	[121]
colorectal cancer	Mitomycin C (MMC) on the day and after surgery + 5-FU for six months vs. MMC + 5-FU as above + PSK (over three years)	disease-free survival (DFS), overall survival (OS)	MMC + 5FU/MMC + 5-FU + PSK	DFS significantly improved in PSK group *p* = 0.013. OS significantly improved in PSK group *p* = 0.013.	448	randomized controlled study	[122]
colorectal cancer	Polysaccharide K(PSK) in combination therapy	relapse-free survival (RFS), overall survival (OS)	UFT + PSK group/UFT group	**RFS:** *Preoperative lymphocyte ratio* < 35%: UFT/PSK vs. UFT 76.5% vs. 55.8% (*p* = 0.008). *Preoperative lymphocyte ratio* ≥ 35% no difference. **OS:** similar to RFS	205 (137/68)	randomized controlled study	[123]
colorectal cancer	FT-207 (750 mgx2/day)/FT-207 + PSK orally (3.0 g/day)	survival	Group A (FT-207)/Group B (FT-207 + PSK orally)	5-year survival rates: Group A vs. B no significant difference.	124 (71/53)	randomized controlled study	[124]
colorectal cancer	UFT/polysaccharide K (PSK)	3-year (DFS, OS)	(UFT/LV)/(UFT/PSK)	3-year DFS/OS: no significant difference	357 (179/ 178)	randomized non-inferiority study	[125]
colorectal cancer	Standard UFT/LV regimen and daily administration of 3 g/day of PSK for 6 months	3-year disease-free survival (DFS)	**Group A:** UFT/LV 6 months **Group B and C:** UFT/LV + PSK (3 g/day) 6 and 12 months	**3-year DFS:** No statistically significant differences among groups **Safety:** *Any-grade AEs*: 59.5% (A), 52.1% (B), 59.2% (C) *Grade* ≥ 3 *AEs*: 13.5% (A), 9.6% (B), 9.9% (C).	186 (37/ 75/74)	randomized phase II trial	[126]

In summary, the applied amount of mushrooms and mushroom extracts in the found clinical trials were as follows: breast cancers < 6 g of powder, <3 g of extract; prostate cancers < 14 g of powder, <5 g of extract; lung cancers < 5.4 g of extract; colorectal cancers < 5.4 g of powder, <3 g of extract. The mushrooms and mushroom extracts were found to be well tolerated but mild side effects may occur.

In patients diagnosed with T2DM the found clinical trials are consistent in that higher than 3 g mushroom, mushroom powder or mushroom extract per day can improve metabolic parameters.

Mushrooms and their extracts in breast cancer patients are similarly well tolerated and also can improve QoL. It can modulate immune function in breast cancer patients; however, it is supposed to have optimal doses which differently modulate the distinct immunological parameters—thus the same dose may activate certain parameters while inhibiting others. Therefore, because these natural components produce more complex effects than expected, cancer patients should be made aware of the fact that these can both enhance and suppress the immune system. The positive effects of mushroom/mushroom extracts on survival in breast cancer patients is more likely subgroup- or marker-specific based on the found clinical trials. Thus, further and more stratified studies are needed to confirm which population of breast cancer patients can benefit from the regular intake of a certain mushroom/extract for their improved survival.

The number of clinical trials with prostate cancer patients in relation to this field is limited to date. The only one randomized and multicenter study investigating survival found beneficial effect of lentinan intake on survival. The other three open-label clinical trials investigating PSA response found no benefit from mushroom powder or extract intake.

In lung cancer patients mushroom extracts significantly improved the QoL. The found clinical trials (with fairly large sample size) were consistent in that beta-glucan administration as complementary therapy can improve survival in lung cancer patients. These results were found both in the cases of specific markers and stage differentiation, but also in general without subgroup analysis. The immunomodulatory role was not confirmed by only one study which investigated this in lung cancer patients; however, this was an open-label study with small sample size.

Based on the result of the clinical trials, using mushroom components may improve QoL in colorectal cancer patients. Immunomodulatory effects—as suggested—depend more on duration rather than the frequency of administration of mushroom or its extracts. The results regarding the survival of colorectal cancer patients are inconsistent, considering that some of the trials with similar study type and comparable sample size found significant improvement in survival time after mushroom or mushroom extract administration while others did not.

A limitation of this review is that there were no sufficient types and/or amount of data to run meta-analysis for quantifying the effects of complementary treatments and publication bias; the outcomes and the set-up of the treatments also differed significantly in most cases. As a general conclusion, trials with lung cancer patients had the biggest sample size, and most of the studies were randomized clinical trials in all the four cancer types, which increases the scientific reliability of these results.

Additionally, to date, there is no FDA approval for mushroom-extracted beta-glucans, triterpenes, or nucleotide analogs for diabetes or cancer treatment. Although cordycepin from *Cordyceps* spp. has promising in vitro anticancer activity, it has limited bioavailability due to rapid in vivo deamination by circulating enzymes, poor uptake into cells and dependency from adenosine kinases for its activation. However, a modified version of this, the ProTide NUC-7738, was in a First-In-Human Phase I Clinical Trial in 2021 [127].

## 6. Conclusions

Edible and medicinal mushrooms contain a wide range of bioactive compounds—including polysaccharides (β-glucans), triterpenes, sterols, phenolic compounds, nucleosides, and enzyme inhibitors—that can modulate metabolic pathways, immune functions, and cellular defense mechanisms in multi-level processes. According to the scientific literature, these compounds have immunomodulatory, anti-inflammatory, antioxidant, insulin-sensitizing, and anti-tumor effects, which establish their application as preventive and complementary therapies in diabetes and cancer.

The collected clinical trials in this review highlighted the benefits of edible/medicinal mushroom or their extracts in QoL and survival in T2DM, breast, lung and colorectal cancer patients. The immunomodulatory properties of these natural ingredients show more complex effects than expected based on results of clinical studies, thus cancer patients should be made aware of the fact that these can both enhance and suppress the immune system. The results suggest that these ingredients are well tolerated even by cancer patients treated with chemotherapy. Although the number of clinical studies are increasing, further human trials are required to clarify optimal dosage and long-term mechanisms of action using similar treatment modalities and study settings to ensure reliable comparisons. However, the results are encouraging in that edible and medicinal mushrooms can be used as preventive and complementary therapies, especially with regard to metabolic and oncological diseases.

## Data Availability

Not applicable.

## Supplementary Materials

The following supporting information can be downloaded at: https://www.mdpi.com/article/10.3390/cancers18142294/s1, Supplementary S1. Main mushroom species, their bioactive compounds, and associated biological effects. (Ref.: references). Supplementary S2. Triterpenes. A. lanostane-type structure. B. Trametenolic acid. C. Ganoderic acid. Supplementary S3. Nucleoside analogues. A. Adenosine, B. Cordycepin, C. Clitocine. Supplementary S4. Clinical trials applying edible/medicinal mushrooms or their extracts at patients diagnosed with impaired glucose tolerance or type 2 diabetes. References [22,23,45,52,57,58,59,64,65,66,71,72,74,75,76,77,78,79,80,81,82,83,128,129,130,131,132,133,134,135,136,137,138,139,140,141,142,143,144] are referring to Supplementary Materials.

## Abbreviations

The following abbreviations are used in this manuscript:

AMPK	AMP activated protein kinase
Aβ	amyloid-β
ABA	anti-beta-glucan antibody
CK	creatinine kinase
COPD	chronic obstructive pulmonary disease
CR3	complement receptor 3
DCR	disease control rate
DFS	disease-free survival
GHS	global health status
GPx	glutathione peroxidase
GSH	glutathione
IAP	immunosuppressive acidic protein
IGT	impaired glucose tolerance
IL-6	interleukin 6
IRS	insulin receptor substrate
LNT	lentinan
MDSCs	myeloid-derived suppressor cells
MMC	mitomycin C
mPFS	median progression-free survival
MST	median survival time
NEFAs	non-esterified free fatty acids
NF-κB	nuclear factor kappa B
NGF	nerve growth factor
NK	natural killer cells
ORR	overall response rate
OS	overall survival
PRR	pattern recognition receptors
PSA	prostate specific antigen
PSK	polysaccharide-K, Krestin
PSP	polysaccharide peptide
QoL	quality of life
RCT	randomized clinical trial
RR	risk ratio
SOD	superoxide-dismutase
T2DM	type 2 diabetes
TCM	Traditional Chinese Medicine
TLR2/6	toll-like receptor 2/6
TNF-α	tumor necrosis factor alpha
WBC	white blood cell
