# Supplementary material for: β-Glucans, Triterpenes and Nucleoside Analogs of Edible and Medicinal Mushrooms as Complementary Treatment in Diabetes and Cancer—A Review of Evidence from Clinical Trials
Source: Cancers (Basel). 2026 Jul 16;18(14):2294. doi: 10.3390/cancers18142294 (PMC13407415; doi:10.3390/cancers18142294)
Supplement: Supplementary file 1 [file cancers-18-02294-s001.zip › cancers-4383296-supplementary.pdf]

Supplementary S1. Main mushroom species, their bioactive compounds, and associated biological effects. (Ref.: references)

**Mushroom species /common name/**	**List of main bioactive compounds**	**List of the known effects**	**Chemical structure**	**Ref.**
Ganoderma lucidum /Lingzhi, Reishi mushroom/	triterpenes /ganoderic acid/, polysaccharides (ß-glucans), glycoproteins (lectines), phenolic compounds, sterols, nucleotides, fatty acids, vitamins, minerals	anti-tumor, immunomodulatory, anti-inflammatory, antiviral (HIV), antimicrobial, antihypertensive effects, cardioprotective, neurotonic, hepatoprotectiv, nephrotonic, antioxidant, antiasthmatic	lanostane structure, β-/1→3/ and /1→6/-glucans, nucleoside and nucleotide structures	[1-4]
Grifola frondosa /Hen-of-the-woods, Maitake/	D-fraction polysaccharides, β-glucans	regulation of blood glucose level, immunostimulation, anti-tumor effect	β-/1→3/, /1→6/-glucan	[5-7]
Lentinula edodes /shiitake/	polysaccharides, β-glucans (lentinan), glycoproteins, phenols, sterols, terpenoids, nucleotides eritadenine	immunomodulatory, anti-tumor, antioxidant, anti-aging, antihypertensive, antiviral, cholesterol-lowering	β-/1→3/, /1→6/-glucan, α-1,4-glucan, lanostan structure, nucleotides, 2(R), 3(R)-dihydroxy-4-(9-adenyl) butyric acid	[8]
Trametes versicolor /Turkey Tail It also known as: Cloud mushroom, Yun Zhi, Kawaritake /	PSK (polysaccharide-K), PSP (polysaccharide-peptide), musarin, vitamin B, fatty acids (linoleic acid, oleic acid, stearic acid, linolenic acid)	immunomodulatory, anticancer, antioxidant, antimicrobial, anti-obesity, antidiabetic, acetylcholinesterase inhibitor	heteropolysaccharides with a β-glucan backbone and protein moieties, (1,3) (1,6)-β-d-glucans, polyphenols (phenolic acids); p-hydroxybenzoic acid, protocatechuic acid, vanillic acid	[9,10]
Hericium erinaceus /Lion's Mane/	Erinacins, hericerins, erinacins M and N, glicoprotein, polysaccharides, β-glucans, sterols, lactone, fatty acids, volatile oil	Neuroprotective, neurodegenerative, cognitive function enhancing, antioxidant, anticancer, anti-aging, immunomodulatory, blood glucose-lowering, cholesterol-lowering	cyathane-type diterpenes, isoindoline-1, hexadecanoic acid, linoleic acid, phenilacetaldehide, benzaldehyde	[7,11-13]
Inonotus obliquus /Chaga/	polysaccharides, fatty acids, hydroxyl acids, polyphenols (phenolic acids, flavonoids, cumarins, quinones), inotodiol, melanines, triterpenoids (lanosterol, trametenolic acid), szteroidok (ergosterol, ergosterol-peroxid)	antioxidant, anti-aging, anti-inflammatory, immunomodulatory, anticancer, antimicrobial, hypoglycaemic, hypolipidemic cardioprotective	lanostane structure, lanosta-8,24-dien-3β,11β-diol	[14-16]

Agaricus blazei Murill /almond mushroom or Himematsutake/	polysaccharides,β-glucans, phenolic and aromatic compounds (vanillic acid, protocatechuic acid, aromatic aldehydes), ergosterol /agarol/, steroid compounds (blazein, volemolide), polyunsaturated fatty acids	immunomodulatory, antiviral, anti-tumor, anti-fatigue activity, antidiabetic, hyperglycaemia effect, anti-inflammatory, anti-carcinogenic, hepatoprotective, antibacterial, anti-mutagenic	β-/1→3/-glucans with β-/1→6/-branches, glycerol monolinoleate, volemolide	[7,17-20]
Pleurotus pulmonarius /oyster mushroom/	polisaccharides (β-D-glucan) ergothioneine, low-molecular-weight phenolic compounds, flavonoids, volatile compounds (aldehydes, alcohols; pentanal, benzaldehyde, hexanol)	antioxidant, anti-inflammatory, blood glucose-lowerint, anti-tumor , antimicrobial, antiproliferative, anti-adhesive	β-glucans /β-/1→3/ and /1→6/-D-glucans/, L-ergothioneine, phenolic compounds such 2-propanediol, and p-anisaldehyde, proteoglycan complexes	[21,22]
Polyporus umbellatus /umbrella polypore /	Polysaccarides (PUP-K), sterols, ergosteroids, triterpenes, volatile phenolic compounds, polyunsaturated fatty acids	immunostimulatory, hepatoprotective, anti-inflammatory, anticancer, antioxidant, hypolipidemic, antimicrobial, anticonvulsant	β-glucans with a β-/1→3/-glucose backbone and β-/1→6/-glucose side chains, polyporusterones A and B (ergostane type), lanostane-type triterpenes with polyphenolic groups, protocatechuic acid, ecdysteroids	[23,24]
Ophiocordyceps sinensis (formerly *Cordyceps sinensis*) /caterpillar fungus/	Cordycepin, cordymin (peptide), adenosine, polysaccharides, β-glucans, trehalose, saponins, polyunsaturated fatty acids (PUFAs), ergosterol, δ-tocopherol, hydroxybenzoicacid	energy-enhancing, immunomodulatory, antioxidant, anti-tumor, hypoglycaemic, hypocholesterolaemia, anti-inflammatory, anti-aging, antimicrobial, anticonvulsant effect, cardioprotective (reduces arrhythmias and chronic heart failure)	cordycepin /3'-dezoxyadenisine/, adenosine, cordycepic-acid (D-mannitol)	[25-31]

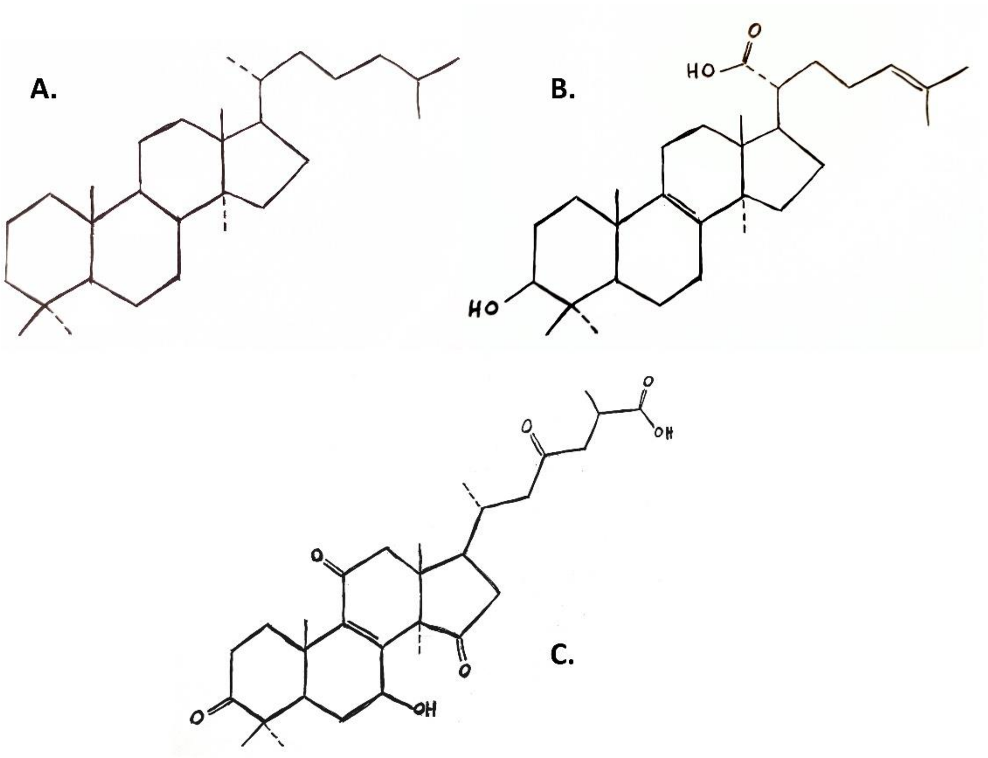

Supplementary S2. Triterpenes. A. lanostane-type structure. B. Trametenolic acid. C. Ganoderic acid.

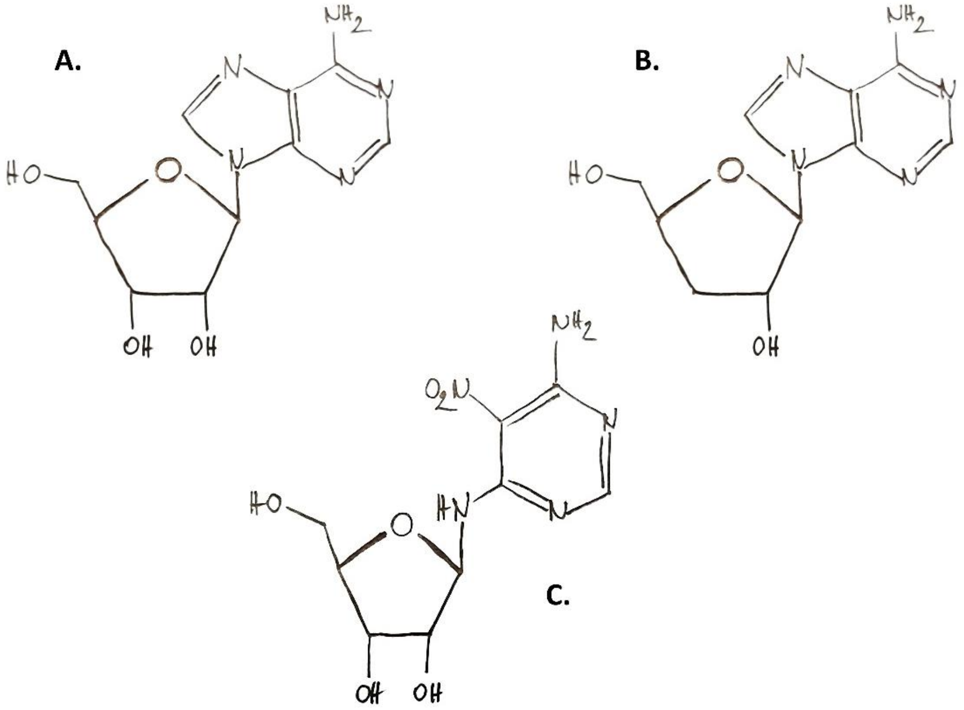

Supplementary S3. Nucleoside analogues. A. Adenosine, B. Cordycepin, C. Clitocine.

Supplementary S4. Clinical trials applying edible/medicinal mushrooms or their extracts at patients diagnosed with impaired glucose tolerance or type 2 diabetes.

Disease state/ type	Complementary treatment(s)	Outcomes	Compared groups	Results	N all (treatment / compared)	Study type	Ref
Impaired glucose tolerance (IGT)	*Pleurotus ostreatus* (20 g powder contains 8.1 g beta-glucans) enriched meal (EN), non-enriched (CON)	glucose and triglyceride responses, and improvement gastrointestinal hormone release	EN/ CON	Non-esterified free fatty acids (NEFAs) under the curve (AUC) decreased by 14% ($p = 0.026$). GLP-1 AUC increase by 17% ($p = 0.001$) EN vs CON. No other significant differences.	22 (11/11)	randomized controlled crossover study	[32]
Healthy volunteers, T2DM	*Pleurotus ostreatus* and *Pleurotus cystidiosus* powder 50 mg/kg/body weight followed by a glucose load	fasting and postprandial glucose and insulin levels	healthy volunteers / type 2 diabetic patients	Healthy volunteers: significant decrease in fasting and postprandial glucose ($P < 0.05$).<br>Type 2 diabetic patients: significant decrease in postprandial glucose ($P < 0.05$) and increased in serum insulin ($P < 0.05$).	50 (22/28)	human study	[33]
T2DM	Ganopoly (polysaccharide fractions extracted from *Ganoderma lucidum*) 1800 mg three times daily orally for 12 weeks	efficacy and safety	Ganopoly / placebo	Ganopoly significantly decreased at week 12: mean HbA1c, mean fasting and postprandial plasma glucose (PPG) levels. No significant change in placebo.<br>Between-groups PPG levels was significant ($p<0.05$) as well as fasting insulin, 2-hour postprandial insulin, fasting C-peptide, 2-hour postprandial C-peptide.<br>Well tolerated.	71	Phase I/II study	[34]
T2DM	diet, exercise + 3 type of biscuits (A: ajwain, B: ajwain+oyster mushroom, C: oyster mushroom) for 3 months	efficacy on glycaemic control, lipid profile and quality of life (QoL).	A / B / C baseline vs post-treatment within the groups	Significant reduction in B and C groups in blood glucose levels ($p<0.005$ both)<br>HbA1c ($p<0.02$ and 0.05), lipid profile ($p<0.001$ and 0.05)<br>QoL improved significantly.	120 (60/60/60)	double-blinded, randomized controlled study	[35]
T2DM	*White Button Mushroom* powder (16 g/day) for 8 weeks	inflammatory and metabolic responses	*WBM* powder/ control	Significant decrease in:<br>fructoseamin ($p = 0.02$)<br>LDL ($p = 0.04$)	44 (22/22)	randomized controlled trial	[36]
T2DM	low-molecular weight hot water extract from *Grifola gargal* 9.2 g daily for 4 weeks	antidiabetic and antiobesity effects	baseline vs post-treatment	Significant decreases in triglyceride levels.	10	clinical trial	[37]
T2DM	*Agaricus blazei Murill* (ABM) extract 1500 mg daily for 12 weeks	HOMA-IR index, insulin resistance	ABM extract / placebo	HOMA-IR after 12 weeks:<br>ABM: 3.6 ± 2.5<br>Placebo: 6.6 ± 7.4<br>($p = 0.04$).<br>Adiponectin change:<br>ABM: +20.0% (±40.7%)<br>Placebo: −12.0% (±20.0%)<br>($p < 0.001$).	60 (29/31)	randomized double-blinded, placebo-controlled clinical trial	[19]

Disease state/ type	Complementary treatment(s)	Outcomes	Compared groups	Results	N all (treatment / compared)	Study type	Ref
T2DM	*Agaricus bisporus* (White Button Mushrooms) consumed 100 g daily for 16 weeks	inflammatory and oxidative stress markers	baseline vs post treatment and baseline vs follow-up (1 month)	Significantly increased the oxygen radical absorbance capacity (p=0.03), adiponectin (p=0.03) at 16 weeks, adiponectin remained unchanged until the end of follow-up - opposite change in advanced glycation end products.	37	retrospecti-ve study	[38]
T2DM	*Ganoderma lucidum* 1.4 g to 3 g /day for 12-16 weeks	blood glucose level, blood pressure and lipid profile	treatment /placebo group	No improvement in plasma glucose, no significant difference in blood pressure or triglycerides.	84	systematic review and meta-analysis of randomized controlled clinical trials	[39]

References

1. Ahmad, M.F. Ganoderma lucidum: Persuasive biologically active constituents and their health endorsement. *Biomed Pharmacother* **2018**, *107*, 507-519, doi:10.1016/j.biopha.2018.08.036.
2. Zeng, P.; Guo, Z.; Zeng, X.; Hao, C.; Zhang, Y.; Zhang, M.; Liu, Y.; Li, H.; Li, J.; Zhang, L. Chemical, biochemical, preclinical and clinical studies of Ganoderma lucidum polysaccharide as an approved drug for treating myopathy and other diseases in China. *J Cell Mol Med* **2018**, *22*, 3278-3297, doi:10.1111/jcmm.13613.
3. Galappaththi, M.C.A.; Patabendige, N.M.; Premarathne, B.M.; Hapuarachchi, K.K.; Tibpromma, S.; Dai, D.Q.; Suwannarach, N.; Rapior, S.; Karunarathna, S.C. A Review of Ganoderma Triterpenoids and Their Bioactivities. *Biomolecules* **2022**, *13*, doi:10.3390/biom13010024.
4. Bryant, J.M.; Bouchard, M.; Haque, A. Anticancer Activity of Ganoderic Acid DM: Current Status and Future Perspective. *J Clin Cell Immunol* **2017**, *8*, doi:10.4172/2155-9899.1000535.
5. Wu, J.Y.; Siu, K.C.; Geng, P. Bioactive Ingredients and Medicinal Values of Grifola frondosa (Maitake). *Foods* **2021**, *10*, doi:10.3390/foods10010095.
6. Konno, S.; Aynehchi, S.; Dolin, D.J.; Schwartz, A.M.; Choudhury, M.S.; Tazaki, H. Anticancer and hypoglycemic effects of polysaccharides in edible and medicinal Maitake mushroom [Grifola frondosa (Dicks.: Fr.) SF Gray]. *International Journal of Medicinal Mushrooms* **2002**, *4*.
7. Hetland, G.; Tangen, J.M.; Mahmood, F.; Mirlashari, M.R.; Nissen-Meyer, L.S.H.; Nentwich, I.; Therkelsen, S.P.; Tjonnfjord, G.E.; Johnson, E. Antitumor, Anti-Inflammatory and Antiallergic Effects of Agaricus blazei Mushroom Extract and the Related Medicinal Basidiomycetes Mushrooms, Hericium erinaceus and Grifola frondosa: A Review of Preclinical and Clinical Studies. *Nutrients* **2020**, *12*, doi:10.3390/nu12051339.
8. Bugajewski, M.; Angerhoefer, N.; Paczek, L.; Kaleta, B. Lentinula edodes as a Source of Bioactive Compounds with Therapeutical Potential in Intestinal Inflammation and Colorectal Cancer. *Int J Mol Sci* **2025**, *26*, doi:10.3390/ijms26073320.
9. Torkelson, C.J.; Sweet, E.; Martzen, M.R.; Sasagawa, M.; Wenner, C.A.; Gay, J.; Putiri, A.; Standish, L.J. Phase 1 Clinical Trial of Trametes versicolor in Women with Breast Cancer. *ISRN Oncol* **2012**, *2012*, 251632, doi:10.5402/2012/251632.
10. He, Z.; Lin, J.; He, Y.; Liu, S. Polysaccharide-Peptide from Trametes versicolor: The Potential Medicine for Colorectal Cancer Treatment. *Biomedicines* **2022**, *10*, doi:10.3390/biomedicines10112841.
11. Ashour, A.; Amen, Y.; Allam, A.; Kudo, T.; Nagata, M.; Ohnuki, K.; Shimizu, K. New isoindolinones from the fruiting bodies of the fungus Hericium erinaceus. *Phytochemistry Letters* **2019**, *32*, 10-14, doi:10.1016/j.phytol.2019.04.017.

12. Tsai-Teng, T.; Chin-Chu, C.; Li-Ya, L.; Wan-Ping, C.; Chung-Kuang, L.; Chien-Chang, S.; Chi-Ying, H.F.; Chien-Chih, C.; Shiao, Y.J. Erinacine A-enriched Hericium erinaceus mycelium ameliorates Alzheimer's disease-related pathologies in APPswe/PS1dE9 transgenic mice. *J Biomed Sci* **2016**, *23*, 49, doi:10.1186/s12929-016-0266-z.
13. Wang, J.; Wu, J.; Yamaguchi, R.; Nagai, K.; Liu, C.; Choi, J.H.; Hirai, H.; Xie, X.; Kobayashi, S.; Kawagishi, H. Uncovering Hericenones from the Fruiting Bodies of Hericium erinaceus through Interdisciplinary Collaboration. *J Nat Prod* **2025**, *88*, 80-85, doi:10.1021/acs.jnatprod.4c01018.
14. Duan, Q.; Tian, L.; Feng, J.; Ping, X.; Li, L.; Yaigoub, H.; Li, R.; Li, Y. Trametenolic Acid Ameliorates the Progression of Diabetic Nephropathy in db/db Mice via Nrf2/HO-1 and NF-kappaB-Mediated Pathways. *J Immunol Res* **2022**, *2022*, 6151847, doi:10.1155/2022/6151847.
15. Lee, M.G.; Kwon, Y.S.; Nam, K.S.; Kim, S.Y.; Hwang, I.H.; Kim, S.; Jang, H. Chaga mushroom extract induces autophagy via the AMPK-mTOR signaling pathway in breast cancer cells. *J Ethnopharmacol* **2021**, *274*, 114081, doi:10.1016/j.jep.2021.114081.
16. Ma, L.; Chen, H.; Dong, P.; Lu, X. Anti-inflammatory and anticancer activities of extracts and compounds from the mushroom Inonotus obliquus. *Food Chem* **2013**, *139*, 503-508, doi:10.1016/j.foodchem.2013.01.030.
17. Huang, K.; El-Seedi, H.R.; Xu, B. Critical review on chemical compositions and health-promoting effects of mushroom Agaricus blazei Murill. *Curr Res Food Sci* **2022**, *5*, 2190-2203, doi:10.1016/j.crfs.2022.10.029.
18. Al-Dbass, A.M.; Al-Daihan, S.K.; Bhat, R.S. Agaricus blazei Murill as an efficient hepatoprotective and antioxidant agent against CCl4-induced liver injury in rats. *Saudi J Biol Sci* **2012**, *19*, 303-309, doi:10.1016/j.sjbs.2012.03.004.
19. Hsu, C.H.; Liao, Y.L.; Lin, S.C.; Hwang, K.C.; Chou, P. The mushroom Agaricus Blazei Murill in combination with metformin and gliclazide improves insulin resistance in type 2 diabetes: a randomized, double-blinded, and placebo-controlled clinical trial. *J Altern Complement Med* **2007**, *13*, 97-102, doi:10.1089/acm.2006.6054.
20. Tangen, J.M.; Tierens, A.; Caers, J.; Binsfeld, M.; Olstad, O.K.; Troseid, A.M.; Wang, J.; Tjonnfjord, G.E.; Hetland, G. Immunomodulatory effects of the Agaricus blazei Murrill-based mushroom extract AndoSan in patients with multiple myeloma undergoing high dose chemotherapy and autologous stem cell transplantation: a randomized, double blinded clinical study. *Biomed Res Int* **2015**, *2015*, 718539, doi:10.1155/2015/718539.
21. Yu, Y.; Liu, T.; Wang, Y.; Liu, L.; He, X.; Li, J.; Martin, F.M.; Peng, W.; Tan, H. Comparative analyses of Pleurotus pulmonarius mitochondrial genomes reveal two major lineages of mini oyster mushroom cultivars. *Comput Struct Biotechnol J* **2024**, *23*, 905-917, doi:10.1016/j.csbj.2024.01.021.
22. Lavi, I.; Levinson, D.; Peri, I.; Tekoah, Y.; Hadar, Y.; Schwartz, B. Chemical characterization, antiproliferative and antiadhesive properties of polysaccharides extracted from Pleurotus pulmonarius mycelium and fruiting bodies. *Appl Microbiol Biotechnol* **2010**, *85*, 1977-1990, doi:10.1007/s00253-009-2296-x.
23. Liu, G.K.; Yang, T.X.; Wang, J.R. Polysaccharides from Polyporus umbellatus: A review on their extraction, modification, structure, and bioactivities. *Int J Biol Macromol* **2021**, *189*, 124-134, doi:10.1016/j.ijbiomac.2021.08.101.
24. Bandara, A.; Rapior, S.; Bhat, D.J.; Kakumyan, P.; Chamyuang, S.; Xu, J.; Hyde, K. Polyporus umbellatus, an Edible-Medicinal Cultivated Mushroom with Multiple Developed Health-Care Products as Food, Medicine and Cosmetics: A Review. *Cryptogamie Mycologie* **2015**, *36*, 3-42, doi:10.7872/crym.v36.iss1.2015.3.
25. Tuli, H.S.; Sandhu, S.S.; Sharma, A.K. Pharmacological and therapeutic potential of Cordyceps with special reference to Cordycepin. *3 Biotech* **2014**, *4*, 1-12, doi:10.1007/s13205-013-0121-9.
26. Tuli, H.S.; Sharma, A.K.; Sandhu, S.S.; Kashyap, D. Cordycepin: a bioactive metabolite with therapeutic potential. *Life Sci* **2013**, *93*, 863-869, doi:10.1016/j.lfs.2013.09.030.

27. Das, G.; Shin, H.S.; Leyva-Gomez, G.; Prado-Audelo, M.L.D.; Cortes, H.; Singh, Y.D.; Panda, M.K.; Mishra, A.P.; Nigam, M.; Saklani, S.; et al. Cordyceps spp.: A Review on Its Immune-Stimulatory and Other Biological Potentials. *Front Pharmacol* **2020**, *11*, 602364, doi:10.3389/fphar.2020.602364.
28. Holliday, J.; Cleaver, M.P. Medicinal value of the caterpillar fungi species of the genus Cordyceps (Fr.) Link (Ascomycetes). A review. *International journal of medicinal mushrooms* **2008**, *10*.
29. De Clercq, E. Curious (old and new) antiviral nucleoside analogues with intriguing therapeutic potential. *Current medicinal chemistry* **2015**, *22*, 3866-3880.
30. Boison, D.; Yegutkin, G.G. Adenosine Metabolism: Emerging Concepts for Cancer Therapy. *Cancer Cell* **2019**, *36*, 582-596, doi:10.1016/j.ccell.2019.10.007.
31. Han, Y.; Dong, C.; Hu, M.; Wang, X.; Wang, G. Unlocking the adenosine receptor mechanism of the tumour immune microenvironment. *Front Immunol* **2024**, *15*, 1434118, doi:10.3389/fimmu.2024.1434118.
32. Dicks, L.; Jakobs, L.; Sari, M.; Hambitzer, R.; Ludwig, N.; Simon, M.C.; Stehle, P.; Stoffel-Wagner, B.; Helfrich, H.P.; Ahlborn, J.; et al. Fortifying a meal with oyster mushroom powder beneficially affects postprandial glucagon-like peptide-1, non-esterified free fatty acids and hunger sensation in adults with impaired glucose tolerance: a double-blind randomized controlled crossover trial. *Eur J Nutr* **2022**, *61*, 687-701, doi:10.1007/s00394-021-02674-1.
33. Jayasuriya, W.J.; Wanigatunge, C.A.; Fernando, G.H.; Abeytunga, D.T.; Suresh, T.S. Hypoglycaemic activity of culinary Pleurotus ostreatus and P. cystidiosus mushrooms in healthy volunteers and type 2 diabetic patients on diet control and the possible mechanisms of action. *Phytother Res* **2015**, *29*, 303-309, doi:10.1002/ptr.5255.
34. Gao, Y.; Lan, J.; Dai, X.; Ye, J.; Zhou, S. A phase I/II study of Ling Zhi mushroom Ganoderma lucidum (W. Curt.: Fr.) Lloyd (Aphyllophoromycetideae) extract in patients with type II diabetes mellitus. *International Journal of Medicinal Mushrooms* **2004**, *6*.
35. Agrawal, R.; Chopra, A.; Lavekar, G.; Padhi, M.; Srikanth, N.; Ota, S.; Jain, S. Effect of oyster mushroom on glycemia, lipid profile and quality of life in type 2 diabetic patients. *Australian Journal of Medical Herbalism* **2010**, *22*, 50-54.
36. Hashemi Yusefabad, H.; Hosseini, S.A.; Zakerkish, M.; Cheraghian, B.; Alipour, M. The effects of hot air-dried white button mushroom powder on glycemic indices, lipid profile, inflammatory biomarkers and total antioxidant capacity in patients with type-2 diabetes mellitus: A randomized controlled trial. *J Res Med Sci* **2022**, *27*, 49, doi:10.4103/jrms.JRMS_513_20.
37. Harada, E.; Morizono, T.; Kanno, T.; Saito, M.; Kawagishi, H. Medicinal Mushroom, Grifola gargal (Agaricomycetes), Lowers Triglyceride in Animal Models of Obesity and Diabetes and in Adults with Prediabetes. *Int J Med Mushrooms* **2020**, *22*, 79-91, doi:10.1615/IntJMedMushrooms.2019033285.
38. Calvo, M.S.; Mehrotra, A.; Beelman, R.B.; Nadkarni, G.; Wang, L.; Cai, W.; Goh, B.C.; Kalaras, M.D.; Uribarri, J. A Retrospective Study in Adults with Metabolic Syndrome: Diabetic Risk Factor Response to Daily Consumption of Agaricus bisporus (White Button Mushrooms). *Plant Foods Hum Nutr* **2016**, *71*, 245-251, doi:10.1007/s11130-016-0552-7.
39. Klupp, N.L.; Chang, D.; Hawke, F.; Kiat, H.; Cao, H.; Grant, S.J.; Bensoussan, A. Ganoderma lucidum mushroom for the treatment of cardiovascular risk factors. *Cochrane Database Syst Rev* **2015**, *2015*, CD007259, doi:10.1002/14651858.CD007259.pub2.
